# Comparison of the Effects of Air Pollution on Outpatient and Inpatient Visits for Asthma: A Population-Based Study in Taiwan

**DOI:** 10.1371/journal.pone.0096190

**Published:** 2014-05-01

**Authors:** Hui-Hsien Pan, Chun-Tzu Chen, Hai-Lun Sun, Min-Sho Ku, Pei-Fen Liao, Ko-Hsiu Lu, Ji-Nan Sheu, Jing-Yang Huang, Jar-Yuan Pai, Ko-Huang Lue

**Affiliations:** 1 Department of Pediatrics, Chung Shan Medical University Hospital, Taichung City, Taiwan R.O.C; 2 School of Medicine, Chung Shan Medical University, Taichung City, Taiwan R.O.C; 3 Department of Health Policy and Management, Chung Shan Medical University, Taichung City, Taiwan R.O.C; 4 Institute of Public Health, Department of Public Health, Chung Shan Medical University, Taichung City, Taiwan R.O.C; University of Tennessee Health Science Center, United States of America

## Abstract

**Background:**

A nationwide asthma survey on the effects of air pollution is lacking in Taiwan. The purpose of this study was to evaluate the time trend and the relationship between air pollution and health care services for asthma in Taiwan.

**Methods:**

Health care services for asthma and ambient air pollution data were obtained from the National Health Insurance Research database and Environmental Protection Administration from 2000 through 2009, respectively. Health care services, including those related to the outpatient and inpatient visits were compared according to the concentration of air pollutants.

**Results:**

The number of asthma-patient visits to health-care facilities continue to increase in Taiwan. Relative to the respective lowest quartile of air pollutants, the adjusted relative risks (RRs) of the outpatient visits in the highest quartile were 1.10 (P-trend  = 0.013) for carbon monoxide (CO), 1.10 (P-trend  = 0.015) for nitrogen dioxide (NO_2_), and 1.20 (P-trend <0.0001) for particulate matter with an aerodynamic diameter ≦10µm (PM_10_) in the child group (aged 0–18). For adults aged 19–44, the RRs of outpatient visits were 1.13 (P-trend = 0.078) for CO, 1.17 (P-trend = 0.002) for NO_2,_ and 1.13 (P-trend <0.0001) for PM_10_. For adults aged 45–64, the RRs of outpatient visits were 1.15 (P-trend = 0.003) for CO, 1.19 (P-trend = 0.0002) for NO_2,_ and 1.10 (P-trend = 0.001) for PM_10_. For the elderly (aged≥ 65), the RRs of outpatient visits in were 1.12 (P-trend  = 0.003) for NO_2_ and 1.10 (P-trend  = 0.006) for PM_10._ For inpatient visits, the RRs across quartiles of CO level were 1.00, 1.70, 1.92, and 1.86 (P-trend  = 0.0001) in the child group. There were no significant linear associations between inpatient visits and air pollutants in other groups.

**Conclusions:**

There were positive associations between CO levels and childhood inpatient visits as well as NO_2_, CO and PM_10_ and outpatient visits.

## Introduction

Asthma is a common chronic inflammatory respiratory disease that affects 300 million people of all ages and all ethnic backgrounds and accounts for about 1 in every 250 deaths worldwide [1]. In Taiwan, the prevalence of asthma increased from 5.07% in 1985 to 11.9% in 2007 [2], [3]. The risk factors for asthma include many external determinants such as mites, dust, mold, indoor and outdoor air pollution, and season variations [4]–[6]. Although air pollution has not been shown as the sole cause of respiratory illnesses, there is evidence that air pollution episodes lead to respiratory irritation, increased use of asthma medications and hospitalizations [7], [8]. Traffic and industry-related pollutants, nitrogen dioxide (NO_2_) and carbon monoxide (CO), were associated with asthma hospitalizations and outpatient visits [9], [10]. Elevated levels of ozone (O_3_), sulfur dioxide (SO_2_) and particulate matter with an aerodynamic diameter ≦10µm (PM_10_) were reported to be related with increased asthma emergency room visits and admissions [11]–[13]. It has been reported that the rise in air pollution has increased respiratory and cardiovascular complications leading to elevated risk of death [14].

If air pollution is responsible for the observed enhanced respiratory complications and mortality, one would also expect to see an impact on clinic visits, outpatient visits, emergency department (ED) visits and hospitalization rates for asthma. However, there are no data regarding a population based survey with seasonal and air pollutants in asthma outpatient and inpatient visits in Taiwan. The objective of this study was to assess asthma-related outpatient and inpatient patterns of visits in different age groups based on the National Health Insurance Research Database (NHIRD) in Taiwan, and to compare the association with utilization of health care services and concentrations of air pollutants.

## Materials and Methods

### Database

The data were obtained from the NHIRD released by the National Health Research Institute (NHRI) in Taiwan. The National Health Insurance Program finances compulsory universal health care for 99% of all of residents of Taiwan [15]. The database contains demographic data, all health-care encounters, expenditure and dates of enrollment and withdrawal. To facilitate research, the NHRI randomly sampled a representative database of one million subjects enrolled in the National Health Insurance program in the year 2005 by a systematic sampling method. This one-million sample was validated to be representative of the entire insured population as reported by the NHRI. The identification numbers and personal information of all individuals in the NHRID were erased to protect the privacy of the individuals. This study was approved by the Institutional Review Board of the Chung-Shan Medical University Hospital, Taiwan.

### Study Population

Cases of asthma were ascertained by the service claim for either outpatient or inpatient visit with a primary diagnosis of asthma (ICD-9-CM code 493.xx). Daily counts of clinic visits, outpatient visits, ED visits and hospital admissions for asthma were extracted from the medical insurance file for the period of 2000–2009. The outpatient visit was defined as a patient visit to a physician's office, clinic, or hospital outpatient department. The inpatient visits include ED visits and admissions. The analyses covered 54 municipalities, each with its own air quality monitoring station. We identified 306628 men and 315178 women who lived in municipalities with air quality monitoring stations during the study period. There were 33934 men and 34527 women with asthma. The patient's exact addresses were not available from the database. Therefore, we assumed that the municipality where a patient visit occurred was regarded as the same area where the patient was most likely exposed to air pollutants. Each asthma outpatient or inpatient visit was matched with the municipality's seasonal average pollutant concentrations. Each occurrence, limited to municipality with air quality monitoring stations, was counted as one visit. The outpatient and inpatient visits were further analyzed by seasons (spring as February, March and April; summer as May, June and July; autumn as August, September and October; winter as November, December and January) and four age groups (0–18, 19–44, 45–64 and ≥65 years).

### Outdoor Air Pollution Monitoring

There were 76 air quality monitoring stations in Taiwan, established by the Environmental Protection Administration. Data from background and industrial air quality monitoring stations were excluded to avoid extreme levels of air pollutants. Finally, data from 54 air quality monitoring stations were enrolled (24 municipalities in northern, 9 municipalities in central, 19 municipalities in southern and 2 municipalities in eastern Taiwan). Complete monitoring data for the air pollutants included PM_10_, SO_2_, CO, O_3_, and NO_2_. The average seasonal concentration of each air pollutant from the Taiwan Environmental Protection Administration's air quality monitoring stations was calculated for further analysis from 2000 to 2009.

### Statistical Analysis

All analyses were done by using the SAS ver. 9.3 software package (SAS Institute, Cary, NC, USA). Multivariate Poisson Regression was made in order to determine the relative risk (RR) of asthma inpatient and outpatient visits by sex, age, year and seasons. For use of health services in relation to air pollutants, the municipalities were defined as the observed units, and the level of air pollutants and counts of patient visits were collected in each municipality for each season from 2000 to 2009. As multiple visits by the same patient in a season in the municipality who lived, the count of the visits was as one visit per season. Generalized estimating equations (PROC GENMOD with repeated statement by the SAS Institute) were used to analyze of levels of pollutants by stratifying municipalities and to compare whether the inpatient and outpatient visits in those municipalities correlated with levels of pollutants. A p value <0.05 was considered statistically significant.

## Results

The descriptive statistics for outpatient and inpatient visits and corresponding period and season data are shown in Table 1. Compared with women, the RRs for inpatient and outpatient visits in men were 1.31 (p<0.0001) and 1.15 (p<0.0001), respectively. Furthermore, when divided into 4 age groups, the highest RRs for inpatient and outpatient visits were among the elderly group (i.e. ≥65 years) followed by the child group (0 –18), the adults aged 45–64 group and the adult aged 19–44 group. For the period effect, there were increased outpatient visits (RR: 1.29, P<0.0001) since 2005 and inpatient visits (RR: 3.30, P<0.0001) since 2006 when compared with patient visits in 2000. The peak seasons for asthma inpatient and outpatient visits in Taiwan were spring (5.1/10,000 person-season) and winter (149.6/10,000 person-season), respectively.

**Table 1:** Inpatient and Outpatient Visits Stratified by Sex, Age, Year and Season in Taiwan.

		Inpatient visits^a^				Outpatient visits^b^			
	Person-seasons	Visits per season	Visit rate per 10000 person-season	RR	*P* value	Visits per season	Visit rate per 10000 person-season	RR	*P* value
Sex									
Female	8297518	2970	3.6	-		104358	125.7	-	
Male	7904684	3711	4.7	1.31	<0.0001	113915	144.0	1.15	<0.0001
Age									
0–18	3930291	2658	6.8	-		93580	237.9	-	
19–44	7373364	1083	1.5	0.22	<0.0001	40932	55.5	0.23	<0.0001
45–64	3674627	1317	3.6	0.53	<0.0001	46489	126.5	0.53	<0.0001
≧65	1223920	1623	13.3	1.96	<0.0001	37272	304.3	1.28	<0.0001
Year									
2000	1611185	369	2.3	-		18604	115.4	-	
2001	1611426	400	2.5	1.08	0.265	19932	123.6	1.07	<0.0001
2002	1628261	393	2.4	1.05	0.469	20624	126.6	1.10	<0.0001
2003	1635688	264	1.6	0.71	<0.0001	19501	119.2	1.03	0.002
2004	1692152	323	1.9	0.83	0.017	23061	136.2	1.18	<0.0001
2005	1719118	239	1.4	0.61	<0.0001	25529	148.4	1.29	<0.0001
2006	1699621	1284	7.6	3.30	<0.0001	24301	143.0	1.24	<0.0001
2007	1685165	1380	8.2	3.58	<0.0001	25208	149.6	1.30	<0.0001
2008	1672283	1212	7.3	3.17	<0.0001	23951	143.2	1.24	<0.0001
2009	1247303	817	6.6	2.86	<0.0001	17562	140.8	1.22	<0.0001
Season									
Spring (Feb-Apr)	4159829	2127	5.1	-		60648	145.7	-	
Summer(May-Jul)	4151745	1416	3.4	0.67	<0.0001	50591	121.8	0.84	<0.0001
Autumn(Aug-Oct)	4140026	1502	3.6	0.71	<0.0001	50887	122.9	0.84	<0.0001
Winter (Nov-Jan)	3750602	1636	4.4	0.85	<0.0001	56147	149.6	1.03	<0.0001

RR, relative risk.

a Inpatient visits include emergency department visits and hospitalizations.

b Outpatient visits include physician's office, clinic, or hospital outpatient department visits.

Table 2 shows the RRs of outpatient health service use with respect to exposure to air pollutants in the four age groups following adjustments for sex, period and quartiles of air pollutants by the generalized estimating equations model. Relative to the respective lowest quartile of air pollutants, the adjusted RRs of the outpatient visits in the highest quartile were 1.10 (95% CI: 1.04, 1.16; P-trend  = 0.013) for CO, 1.10 (95% CI: 1.01, 1.18; P-trend  = 0.015) for NO_2_, 0.94 (95% CI: 0.88, 0.99; P-trend  = 0.016) for O_3_, and 1.20 (95% CI:1.13, 1.27; P-trend <0.0001) for PM_10_ in the child group. For adults aged 19–44, the adjusted RRs of the outpatient visits were 1.13 (95% CI: 1.03, 1.23; P-trend = 0.078) for CO, 1.17 (95% CI: 1.05, 1.31; P-trend = 0.002) for NO_2,_ 0.88 (95% CI: 0.83, 0.94; P-trend<0.0001) for O_3_, and 1.13 (95% CI: 1.05, 1.21; P-trend <0.0001) for PM_10_. The adjusted RRs of the outpatient visits in the adults aged 45–64 were 1.15 (95% CI: 1.05, 1.26; P-trend = 0.003) for CO, 1.19 (95% CI: 1.08, 1.30; P-trend = 0.0002) for NO_2,_ 0.93 (95% CI: 0.89, 0.98; P-trend = 0.028) for O_3_, and 1.10 (95% CI: 1.02, 1.18; P-trend = 0.001) for PM_10_. The adjusted RRs for the elderly outpatient visits across quartiles of air pollutant level were 1.00, 1.02, 1.07, and 1.12 (P-trend  = 0.003) for NO_2_ and 1.00, 1.04, 1.06, 1.10 (P-trend  = 0.006) for PM_10._ There was no significant association between SO_2_ and outpatient visits in any group.

**Table 2:** Outpatient Visits in Relation to Air Pollutants by Different Age Groups.

	Outpatient visits^a^							
	Age group							
	0–18		19–44		45–64		≥65	
Variables	RR	95%CI	RR	95%CI	RR	95%CI	RR	95%CI
Men/women	**1.43**	1.37–1.48	**0.84**	0.79–0.90	**0.77**	0.72–0.82	**1.20**	1.12–1.29
Year	**1.06**	1.05–1.07	**1.03**	1.02–1.04	1.00	0.99–1.02	1.01	0.99–1.02
CO (ppm)								
0.19–0.43	-		-		-		-	
0.43–0.55	**1.07**	1.03–1.11	**1.13**	1.07–1.20	**1.06**	1.01–1.12	1.02	0.97–1.06
0.55–0.69	**1.06**	1.01–1.12	**1.12**	1.03–1.21	**1.11**	1.04–1.20	1.02	0.96–1.09
0.69–1.22	**1.10**	1.04–1.16	**1.13**	1.03–1.23	**1.15**	1.05–1.26	1.04	0.95–1.13
P-Trend	**0.013**		0.078		**0.003**		0.450	
NO_2_ (ppm)								
4.45–14.01	-		-		-		-	
14.01–18.64	1.02	0.97–1.08	1.03	0.95–1.11	**1.07**	1.01–1.14	1.02	0.97–1.07
18.64–24.04	1.05	0.98–1.12	1.08	0.98–1.19	**1.14**	1.06–1.23	**1.07**	1.00–1.13
24.04–47.84	**1.10**	1.01–1.18	**1.17**	1.05–1.31	**1.19**	1.08–1.3	**1.12**	1.03–1.21
P-Trend	**0.015**		**0.002**		**0.0002**		**0.003**	
O_3_ (ppm)								
14.23–23.83	-		-		-		-	
23.83–27.23	0.99	0.96–1.03	**0.96**	0.93–0.99	**0.96**	0.93–0.99	0.98	0.94–1.02
27.23–30.80	0.96	0.92–1.00	**0.94**	0.90–0.98	0.98	0.94–1.01	0.99	0.94–1.04
30.80–50.00	**0.94**	0.88–0.99	**0.88**	0.83–0.94	**0.93**	0.89–0.98	**0.95**	0.90–0.99
P-Trend	**0.016**		**<0.0001**		**0.028**		0.103	
PM_10_ (μg/m^3^)								
23.00–43.67	-		-		-		-	
43.67–54.00	**1.06**	1.02–1.10	**1.07**	1.03–1.11	1.02	0.98–1.07	**1.04**	1.01–1.08
54.00–71.00	**1.11**	1.06–1.17	**1.14**	1.09–1.19	**1.09**	1.03–1.14	**1.06**	1.01–1.11
71.00–141.67	**1.20**	1.13–1.27	**1.13**	1.05–1.21	**1.10**	1.02–1.18	**1.10**	1.03–1.17
P-Trend	**<0.0001**		**<0.0001**		**0.001**		**0.006**	
SO_2_ (ppm)								
0.20–2.80	-		-		-		-	
2.80–3.77	1.03	0.97–1.08	0.99	0.93–1.05	1.00	0.94–1.05	1.04	0.99–1.09
3.77–5.00	1.05	0.98–1.11	0.96	0.90–1.03	0.96	0.90–1.03	1.05	0.99–1.10
5.00–19.17	1.04	0.97–1.13	0.96	0.89–1.04	0.97	0.90–1.05	1.01	0.95–1.08
P-Trend	0.201		0.216		0.340		0.590	

Abbreviation: CO, carbon monoxide; NO_2_, nitrogen dioxide; O_3_, ozone; PM_10_, particulate matter with an aerodynamic diameter ≦10µm; RR, relative risk; SO_2_, sulfur dioxide.

a Outpatient visits include physician's office, clinic, or hospital outpatient department visits.

The use of inpatient health services in relation to levels of air pollutants by different age groups is shown in Table 3. The adjusted RRs for the child inpatient visits across quartiles of CO level were 1.00, 1.70, 1.92, and 1.86 (P-trend  = 0.0001) after multivariate adjustment by sex, year and other air pollutants. There were no significant linear associations between inpatient visits and air pollutants in other age groups.

**Table 3:** Inpatient Visits in Relation to Air Pollutants by Different Age Groups.

	Inpatient visits^a^							
	Age group							
	0–18		19–44		45–64		≥65	
Variables	RR	95%CI	RR	95%CI	RR	95%CI	RR	95%CI
Men/women	**1.86**	1.67–2.07	0.98	0.83–1.16	0.82	0.66–1.01	1.14	0.96–1.34
Year	**1.34**	1.28–1.39	**1.22**	1.17–1.27	**1.12**	1.08–1.15	**1.15**	1.10–1.20
CO (ppm)								
0.19–0.43	-		-		-		-	
0.43–0.55	**1.70**	1.40–2.06	1.08	0.83–1.40	1.17	0.92–1.49	1.28	0.98–1.66
0.55–0.69	**1.92**	1.53–2.40	1.07	0.81–1.42	1.18	0.90–1.55	1.35	0.93–1.97
0.69–1.22	**1.86**	1.39–2.48	0.94	0.64–1.38	1.21	0.87–1.68	1.42	0.94–2.15
P-Trend	**0.0001**		0.856		0.343		0.106	
NO_2_ (ppm)								
4.45–14.01	-		-		-		-	
14.01–18.64	0.85	0.70–1.03	0.86	0.65–1.13	0.85	0.68–1.07	1.09	0.85–1.40
18.64–24.04	0.85	0.66–1.08	1.08	0.75–1.55	1.09	0.83–1.44	1.22	0.85–1.76
24.04–47.84	0.90	0.67–1.20	1.33	0.92–1.94	1.08	0.76–1.53	1.17	0.77–1.76
P-Trend	0.615		0.068		0.447		0.434	
O_3_ (ppm)								
14.23–23.83	-		-		-		-	
23.83–27.23	**0.86**	0.74–0.99	1.02	0.90–1.16	1.00	0.81–1.23	1.14	0.99–1.31
27.23–30.80	0.87	0.74–1.02	1.08	0.87–1.32	1.13	0.88–1.44	**1.18**	1.01–1.39
30.80–50.00	0.83	0.66–1.05	1.04	0.79–1.35	1.06	0.80–1.40	1.20	0.99–1.45
P-Trend	0.136		0.548		0.450		0.070	
PM_10_ (μg/m^3^)								
23.00–43.67	-		-		-		-	
43.67–54.00	**0.84**	0.73–0.97	1.04	0.83–1.30	**0.82**	0.69–0.97	0.89	0.74–1.07
54.00–71.00	0.97	0.83–1.13	1.25	0.96–1.63	1.01	0.82–1.22	1.00	0.81–1.23
71.00–141.67	0.89	0.71–1.12	1.05	0.77–1.44	1.04	0.78–1.37	0.93	0.70–1.23
P-Trend	0.757		0.336		0.393		0.900	
SO_2_ (ppm)								
0.20–2.80	-		-		-		-	
2.80–3.77	0.95	0.77–1.18	1.13	0.83–1.52	0.90	0.69–1.17	0.95	0.72–1.26
3.77–5.00	1.03	0.79–1.33	1.26	0.96–1.65	0.91	0.70–1.19	1.00	0.76–1.31
5.00–19.17	0.82	0.63–1.07	0.96	0.71–1.29	0.81	0.61–1.08	0.78	0.57–1.05
P-Trend	0.369		0.882		0.204		0.223	

Abbreviation: CO, carbon monoxide; NO_2_, nitrogen dioxide; O_3_, ozone; PM_10_, particulate matter with an aerodynamic diameter ≦10µm; RR, relative risk; SO_2_, sulfur dioxide.

a Inpatient visits include emergency department visits and hospitalizations.

**Figure 1:**
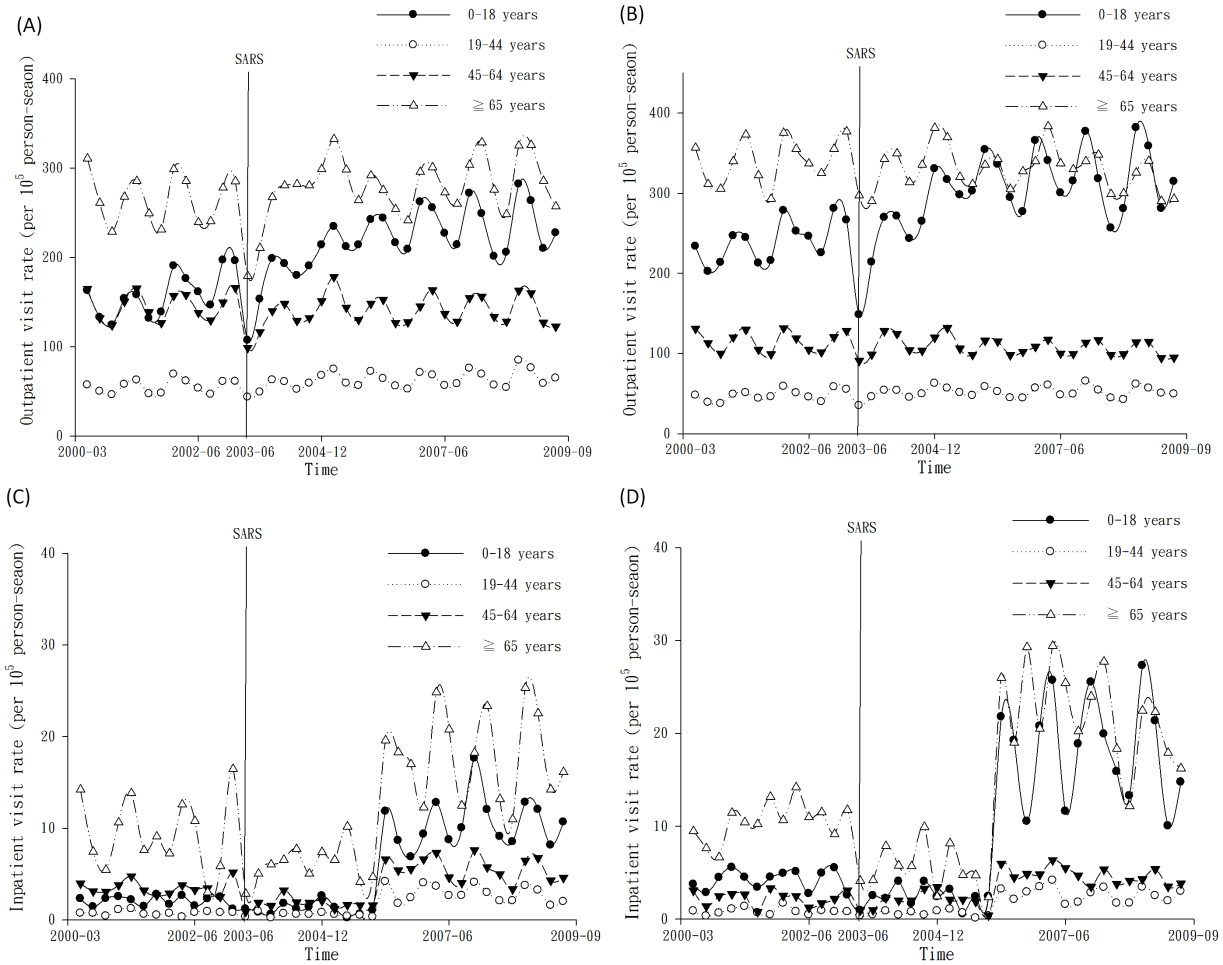
Outpatient visit rates for asthma in 4 age groups in women (A) and men (B), and inpatient visit rates in 4 age groups in women (C) and men (D) during 2000–2009. SARS, Severe Acute Respiratory Syndrome.

The use of inpatient health services increased with time as shown in Fig 1. In general, there were trends of increasing asthma outpatient visits in the children's group (Fig. 1A & B). In 2003, there was an outbreak of Severe Acute Respiratory Syndrome (SARS) in Hong Kong and nearly became a pandemic event [16]. During this period, inpatient visits dramatically decreased in all age groups. After the SARS outbreak, the rates of inpatient visits have increased with time since 2006, especially in the child and the elderly groups (Fig 1C & D).

## Discussion

Among patients with asthma, air pollutant exposure causes increased asthma morbidity. Little is known about changes over time in air pollutant exposure among patients with asthma in a national sample. During the study period, the inpatient and outpatient visits by men were higher than in women. The peak seasons of asthma inpatient and outpatient visits for the total population were spring and winter, respectively. The inpatient and outpatient visits of asthmatics have not reached a plateau and have continued to increase. Inpatient visits for asthma increased with increased levels of CO in children but not for any pollutants in adults in the present study. Our study found that CO, NO_2_ and PM_10_ had significant estimated associations on outpatient visits due to asthma and children are more susceptible than other age groups.

Health care visits only illustrate a small percentage and most severe inpatients of the total impacts of air pollution. Our study used the count of health care visits from NHRI databases as the measure of morbidity in the population. Clinic visits, outpatient visits, ED visits and admissions are types of health care service, but also possessed of potentially important disparities [17], [18]. Because asthma is a chronic disease, patients with asthma were taught to deal with their symptoms and discomfort of an asthma attack [19], [20]. When the concentrations of air pollutants rise, patients with asthma may have treated symptoms by themselves or visited neighborhood clinics and hospital outpatient departments for medical treatment. Subsequently, if patients did not receive any treatment or if the condition was deteriorated or ineffective after an outpatient visit, they would then visit hospital emergency departments for assistance. This may explain why there was no increased risk for inpatient visits for adults with increasing levels of air pollutants.

In response to the increasing prevalence, mortality rates, and medical cost of asthma, the Bureau of National Health Insurance initiated a Healthcare Quality Improvement Program for patients with asthma since November, 2001 [21]. The characteristics of the program were registry development, adherence to guidelines, patient education, and nursing care management. The Bureau offered financial incentives that motivated the nurses and physicians to change their practice patterns by following clinical guidelines; thereby, the asthma care team support from healthcare organizations was able to promote and enable patients to effectively self-manage their asthma with reduced healthcare resource utilization. During the SARS outbreak, people avoided hospital visits to prevent themselves from nasocomial infection and the inpatient visit rate for asthma decreased in all age groups [22]. However, there was an increase of inpatient and outpatient visits after the SARS outbreak. The exacerbation of air pollution probably plays a role in the rising rate of asthma visits due to growing populations, increased economic activity, rise in vehicular traffic, as well as the increasing intensity and occurrence of dust storms originating in Mongolia and China [23].

There have been associations between daily ambient air pollution levels and acute exacerbations of respiratory diseases in many time-series studies [11], [12], [24]. Urban air pollution constitutes a complex mixture of several compounds. The exposure of motor vehicle air pollutants, such as NO_2_, CO, SO_2_ and PM, increases the incidence and prevalence of asthma and bronchitic symptoms [25]. CO was reported to be associated with asthma admission and ED visits [9], [26], [27]. There were associations between short-term exposure to ambient CO and risk of cardiovascular disease hospitalizations, even at low ambient CO levels [28]. Sun *et al* conducted a one-year observation in central Taiwan that CO played a role in acute exacerbation of asthma in children and increased the number of childhood asthma ED visits, but not in adults [29]. Villeneuve et al reported that an increase in the interquartile range of the 5-day average for CO was associated with 48% increases in the risk of an asthma ED visit for children aged 2 to 4, but the associations were less pronounced in adult aged 15 to 44 [30]. In Rome, where air pollution comes mostly from combustion products of motor vehicles, CO was associated with most of the respiratory conditions in all ages, and it remained an independent predictor in multi-pollutant analysis for all respiratory admissions [31]. In London, there were significant associations between CO and daily consultations for asthma and other lower respiratory disease in children, whereas in adults the only consistent association was with PM_10_ [32]. In our study, CO was also significantly associated with asthma exacerbation and inpatient visits in children. However, a direct association between CO and asthma lacks a biologically plausible mechanism [33]. It is possible that CO might be a surrogate for other noxious incomplete combustion products [34]. Unlike with children, the other major confounders in adult asthmatic patients include occupational exposures, smoking, stress, emotional factors, and systemic diseases, which may also partly explain why outdoor air pollution was not associated with inpatient visits in the adults in our study.

Coal- and oil-fired power plants and diesel- and gasoline-powered motor vehicle engines are the main sources of ambient NOx emissions [35]. In a meta-analysis study, inhalation of NO_2_ in the air significantly increased the development of childhood asthma and symptoms of wheezing [36]. A previous study in Taipei showed that most robust associations were found for NO_2_ elevation and asthma admission rates [26]. NO_2_ levels were associated with childhood asthma exacerbations and ED visits in Santa Clara, California [37]. In a spatiotemporal analysis of air pollution and asthma patient visits in Taipei, elevated levels of NO_2_ had a positive association on outpatient visits [38]. In summary, there was a linear response in outpatient visits on days with elevated NO_2_.

Previous studies have not yielded consistent results concerning associations between O_3_ and SO_2_ and asthma admissions. SO_2_ was least frequently mentioned in the correlation with asthma hospitalization rate. Most previous studies have not shown a significant effect of SO_2_ on asthma hospitalization rates supporting our findings [29], [39]. O_3_ is a highly reactive gas and induces bronchial inflammation, constriction of the airways and decreased lung function [40]. Long-term exposure to outdoor O_3_ increases the prevalence of bronchitic symptoms among children [41]. O_3_ levels have been previously reported to be associated with asthma admission and ED visits [11], [24], [38]. In contrast, asthma exacerbations were not associated with O_3_ levels in North America [42]. Daily general-practice consultations for respiratory conditions were unrelated to O_3_ in London [32] and Taiwan [43].

PM_10_ is a heterogeneous mixture of small solid or liquid particles with varying compositions in the atmosphere. There were no consistent results concerning associations between PM_10_ and asthma admissions. Some studies reported that PM_10_ was significantly associated with asthma admissions [8], [39], other studies reported a lack of association between PM_10_ and asthma admission [27]. In our study, levels of PM_10_ were associated with outpatient visits for asthma, but not associated with admissions. Hwang and Chan used data obtained from clinic records and environmental monitoring stations in Taiwan and reported that PM_10_ had an impact on outpatient visits [43]. This was consistent with our findings.

Most of the previous studies are cross-sectional and have focused air pollutants on short-term, regional area and respiratory system diseases [8], [29], [38]. We conducted a nationwide asthma survey on the effects of air pollution in Taiwan and evaluated the association between different air pollutants and outpatient and inpatient visits. One of the strengths of the present study is the use of a computerized database, which is population-based and is highly representative. Because we enrolled all patients diagnosed with asthma from 2000 to 2009, we can rule out the possibility of selection bias. Since the data were obtained from a historical database, which collects all information, recall bias was avoided. Besides, we analyzed levels of pollutants at different municipalities and compared whether the inpatient and outpatient visits in those municipalities correlated with levels of pollutants. Thus this study directly associated the patient visits with the levels of pollutants in those municipalities. As multiple visits by the same patient would lead to misinterpretation of the data, we used the statistic method to reduce bias.

There were still several limitations of the present study. First, although we adjusted for several potential confounders in the statistical analysis, a number of possible confounding variables, including family history of atopy, dietary habits, physical activity, occupational exposures, smoking habits, stress and emotional factors, which are associated with asthma were not included in our database. Second, we were unable to ask patients for severity of asthma because of de-identifcation. Third, self-treatment with over-the-counter medications or alternative health services was not included in the database. These data also do not include the number of asthmatic subjects who had respiratory problems but did not search for health service. Therefore, the extent of the issue may have been considerably underestimated. Fourth, potentially inaccurate data in the records could lead to possible mis-classification.

In conclusion, the present study provides evidence that exposure to the outdoor air pollutant, CO, exerted adverse effects on health and increases in the child admission. Our study also showed a linear association between NO_2_, CO, and PM_10_ and outpatient visits. It is an important public health policy to monitor air quality and warn the public about atmospheric factors that could be associated with increased risks of asthma.
